# Effect of Cigarette Smoke Extract on Dendritic Cells and Their Impact on T-Cell Proliferation

**DOI:** 10.1371/journal.pone.0004946

**Published:** 2009-03-18

**Authors:** Esmaeil Mortaz, Aletta D. Kraneveld, Joost J. Smit, Mirjam Kool, Bart N. Lambrecht, Steven L. Kunkel, Nicholas W. Lukacs, Frans P. Nijkamp, Gert Folkerts

**Affiliations:** 1 Division of Pharmacology and Pathophysiology, Utrecht Institute for Pharmaceutical Sciences, Utrecht University, Utrecht, The Netherlands; 2 Department of Biochemistry, Faculty of Medical Sciences, Tarbiat Modarres University, Tehran, Iran; 3 Department of Basic Science, Section of Biochemistry, Faculty of Veterinary Medicine, Urmia University, Urmia, Iran; 4 Institute for Risk Assessment Sciences (IRAS), Utrecht University, Utrecht, The Netherlands; 5 Department of Pulmonary Medicine, Erasmus University Medical Center, Rotterdam, The Netherlands; 6 Laboratory of Immunoregulation and Mucosal Immunity, Department of Pulmonary Medicine, Ghent University, Ghent, Belgium; 7 Department of Pathology, University of Michigan Medical School, Ann Arbor, Michigan, United States of America; Institut de Pharmacologie et de Biologie Structurale, France

## Abstract

Chronic obstructive pulmonary disease (COPD) is characterized by chronic airway inflammation. Cigarette smoke has been considered a major player in the pathogenesis of COPD. The inflamed airways of COPD patients contain several inflammatory cells including neutrophils, macrophages,T lymphocytes, and dendritic cells (DCs). The relative contributions of these various inflammatory cells to airway injury and remodeling are not well documented. In particular, the potential role of DCs as mediators of inflammation in the smoker's airways and COPD patients is poorly understood. In the current study we analyzed the effects of cigarette smoke extract on mouse bone marrow derived DC and the production of chemokines and cytokines were studied. In addition, we assessed CSE-induced changes in cDC function in the mixed lymphocyte reaction (MLR) examining CD4+ and CD8+ T cell proliferation. Cigarette smoke extract induces the release of the chemokines CCL3 and CXCL2 (but not cytokines), via the generation of reactive oxygen species (ROS). In a mixed-leukocyte reaction assay, cigarette smoke-primed DCs potentiate CD8^+^T cell proliferation via CCL3. In contrast, proliferation of CD4^+^T cells is suppressed via an unknown mechanism. The cigarette smoke-induced release of CCL3 and CXCL2 by DCs may contribute to the influx of CD8^+^T cells and neutrophils into the airways, respectively.

## Introduction

Chronic Obstructive Pulmonary Disease (COPD) is a multicomponent disease characterize by emphysema and/or chronic bronchitis [1]. The pulmonary component is characterized by airflow limitation that is not fully reversible. The airflow limitation is usually progressive and associated with an abnormal inflammatory response of the lung to noxious particles or gases [2].

COPD is mostly associated with cigarette smoking and thereby cigarette smoke is defined as a major risk factor [3]. Several inflammatory cells and their mediators, both of the innate and adaptive immune system, participate in the inflammatory response in COPD., Macrophages, neutrophils and CD8+ T cells are the cells usually considered the prime effector cells in pathogenesis of COPD [4], but recently DCs have been suggested to be a potentially important new player/orchestrator of the pattern of inflammation that characterizes of COPD [5].

In both humans and mice there are several subtypes of DCs, as characterized by surface markers and function. Generally, DCs can be distinguished into conventional DCs (cDCs) and plasmacytoid DCs (pDCs) [6]–[8] . cDCs are crucial antigen-presenting cells (APCs) for primary T-cell responses. They arise from bone marrow (BM) precursors that colonize peripheral tissues through the blood or lymph [9]. In vitro studies using bone marrow and monocyte-derived DCs exposed to varying doses of nicotine [10], [11] and cigarette smoke extract (CSE) [11] have yielded contrasting results with respect to their effect on DC function.

cDCs might play a central role in bridging innate and adaptive immunity via direct cell-cell interactions and/or cytokine production [12], [13]. These interactions may influence the activation status of cells from the adaptive immune system such as CD4^+^T cells and CD8^+^T cells [5], [7], [13]–[15] CD8^+^T cells could be essential for the development of cigarette smoke-induced COPD [12]. In this context, it has been reported that cigarette smoke in humans reduces DC maturation and function. Changes that favor repeated infection, increased exacerbation frequency, and the altered (CD8^+^T-cell predominant) pattern of inflammation associated with this progressive chronic disease [15]. Moreover, Robbins et al provided evidence that cigarette smoke exposure causes specific defects in DC maturation and suppresses the proliferation of CD4^+^T cells in thoracic regional lymph nodes in mice [13].

To investigate the effect of cigarette smoke on cDC, these cells were incubated with CSE and different chemokines and cytokines were measured and accordingly the molecular mechanisms were studied. In addition, we assessed CSE-induced changes in cDC function in the mixed lymphocyte reaction (MLR) examining CD4+ and CD8+ T cell proliferation.

## Materials and Methods

### Reagents

GM-CSF was purchased from PeproTech (London, UK). Trizol and SuperScript II were purchased from Invitrogen (CA, USA). Sybrgreen Universal PCR Master Mix was obtained from ABgene (Hamburg, Germany). LPS, propidium ionide (PI), N-acetylcysteine (NAC), SB 239063, and curcumin were obtained from Sigma-Aldrich (Zwijndrecht, The Netherlands). The CCL3, CXCL2, MCP-1, KC ELISA kits, neutralizing antibodies for CCL3 and CXCL2 were purchased from R&D systems (Oxon, UK). Mouse inflammatory and Th1/Th2 cytokine beads array (CBA) kits, annexin V, 7-AAD were purchased from BD (Alphen, The Netherlands). Rabbit polyclonal antibody against IκB-α and p65 were obtained from Santa Cruz Biotechnology (Heerhugowaard, The Netherlands). Mouse monoclonal antibodies specific for JNK/SAPK, phospho-Erk1/2, β-actin, phospho p38, p38, phospho c-jun and c-jun were obtained from Cell Signaling (Leiden, The Netherlands). Functional Grade Purified anti-mouse Toll-like receptor 4 (TLR4)/MD-2 (Clone: MTS510 0) and isotype control (Rat IgG2a, κ) were purchased from ebioscience (San Diego, CA, USA). ATF-2 and c-fos and lamin C were obtained from Stressgen (Uden, The Netherlands). Horseradish peroxidase (HRP)-conjugated rabbit-anti mouse IgG, mouse anti-rabbit and goat anti-rabbit IgG were purchased from Dako (Heverlee, Belgium). A nuclear and cytoplasmic extraction kit, super blocking buffer and bicinchoninic acid (BCA) protein assay kit were purchased from Pierce (Amsterdam, The Netherlands). CFSE dye and miniTM protease inhibitors were obtained from Molecular Probes (Eugene, OR, USA) and Roche (Almere, The Netherlands), respectively.

### Experimental animals

Ten- to 12-week-old Balb/c or C57BL/6 and MyD88 knockout mice (kindly provided by Dr. S. Kunkel) were purchased from The Jackson Laboratory (ME, USA) and maintained in the pathogen-free Central Animal Facility of the University of Utrecht and University of Michigan. All experiments were approved by the University Utrecht and University of Michigan Committee on the Use and Care of Animals.

### Preparation of Cigarette Smoke Extract (CSE)

CSE was produced following the method as described before [16]. Nontoxic concentrations of CSE were assayed performing toxicological assays (lactase dehydrogenase) and flow cytometery analysis (annexin-V and 7-AAD staining). We also performed a dose–response to establish the effect of different CSE concentrations on chemokine and cytokine release of cDCs. No toxic effects of up to 1.5% concentration of CSE was found since viability was consistently established to be >95% (trypan blue exclusion).

### Generation of bone marrow dendritic cells culture with GM-CSF

The method for generating BM-derived cDCs was modified (for higher purity) from that described originally by Inaba and coworkers [17].

### Cell activation

Cells at 9 days of culture were washed and pre-incubated with pharmacological inhibitors for 30 min, and then stimulated with CSE (1.5%) or LPS (100 ng/ml, positive control) for 30 min for protein expression in cytoplasmic and nuclear fraction, for determination of chemokines at mRNA or protein levels by ELISA and Real time-PCR, at 4 and 16 h, respectively. For MLR, cDC were incubated with CSE (1.5%) for 24 h and then washed and co-cultured with CD8^+^ and CD4^+^T cells for 72 h.

### Chemokines and cytokine assays

CCL3, CXCL2, MCP-1 and KC at protein concentrations in supernatants of cells were quantified using ELISA kits according to the manufacturer's instructions. To quantify the inflammatory cytokines (TNF-α, IL-2, IL-6, IL-10, IL-12p70, MCP-1, IFN-γ), 50 µl of culture medium were subjected to CBA kits by using FACS analysis according to the manufacturer's instruction.

### RNA isolation and real time PCR

Total RNA was extracted from cDCs by using Trizol according to standard protocols. Reverse transcription was performed with SuperScript II. For real-time RT-PCR, cDNA was analyzed for the expression of CCL3, CXCL2 and GAPDH/B2M genes using Sybrgreen using an ABI Prism 7000 Sequence Detection System (Applied Biosystems) under conditions of 50°C for 2 minutes, 95°C for 10 minutes, then 40 cycles of 95°C for 15 seconds and 60°C for 1 minute. The sequences for PCR primers (Eurogentec) were used as described before [18], [19].

### Measurement of intracellular ROS

Intracellular ROS levels were measured by flow cytometry in cells cultured in serum-free medium and loaded with the redox-sensitive dye DCFH-DA (D399) [20]. Thirty minutes before the end of each incubation period, cells were incubated with 10 µM DCFH-DA in dark. Cells were thoroughly and quickly washed with PBS and immediately acquired for analyzed for fluorescence as described before [20], [21] by FACSCalibur (BD Bioscienes). The data were plotted and analyzed using CellQuest software. PMA at concentration 0.1 µg/ml used as a positive control.

### Preparation of cytoplasmic and nuclear extracts

Cells were washed twice with PBS and layzed with cytoplasmic extraction reagent containing protease inhibitors (MiniTM protease inhibitors, cocktail).as described before [22]. Protein concentrations were determined by using a bicinchoninic acid (BCA) protein assay kit (Pierce).

### Western blot analysis

After activation, cDC were washed once with PBS and lysed in lysis buffer containing MiniTM protease inhibitors. The lysate (25 or 50 µg) was subjected to SDS/PAGE [10% (w/v) gel] and blotteing as described before [22]. After blocking the membranes with blocking buffer, the membranes were probed with antibodies in recommended concentration as described in usage instruction antibodies. After three washes with TBS-T, membranes were treated for 1 h with HRP-conjugated indicated antibodies diluted to 1∶20,000 in TBS-T. After three washes with TBS-T, immunoreactive protein bands were revealed with an ECL and ECL Plus (Amersham). Films were scanned and analyzed on a GS7-10 Calibrated Imaging Densitometer equipped with Quantity One v. 4.0.3 software (Bio-Rad).

### Quantification of AP-1 and NF-κB activities

We analyzed NF-κB and AP-1 activation by using the Trans-AM NF-κB p65/NF-κB p50 and AP-1 Transcription Factor Assay Kit (Active Motif, Rixensart), respectively, according to the manufacturer's instructions.

### Mixed lymphocyte reaction (MLR)

cDCs (from Balb/c mice) at day 8 were pretreated with CSE (1.5%) for 24 h and then used to stimulate CD4^+^T cells or CD8^+^T cells (from C57BL/6 mice) . The MLR was conducted in round-bottom; 96-well micro test plates in 0.2 ml of RPMI with (10%) FCS in the continued presence of the blocking mAb at 20 µg/ml. Graded doses of cDCs were added as indicated in *Results* *section*. To monitor the MLR, the CD4^+^T cells or CD8^+^T cells were isolated from spleen by using CD4^+^ and CD8^+^T cell isolation kits (Miltenyibiotec) and then cells (5×10^7^/ml) loaded with the proliferation-tracking dye CFSE at a concentration of 4 µM in phosphate-buffered saline for 15 min at 37°C. Labelled cells were then washed three times. The MLR was assessed by CFSE dilution after 72 h after co-culturing with T cells by FACS analysis. For FACS analysis, cells were washed and labelled with CD3 conjugated with APC, CD4^+^ and CD8^+^ conjugated with PE antibodies plus PI for 30 min. Then after 2 times washing with FACS buffer (PBS, 5% FCS, 0.1% sodium azide) the proliferation of T cells were measured by flow cytometery. T To determine the production of IL-2 induced by CSE-conditioned cDCs, supernatants of T cells were harvested for measurement of IL-2.

### Application of neutralizing antibodies

Using neutralizing antibodies directed against CCL3 or CXCL2, we investigated the role of these chemokines in MLR response of CSE-primed cDC. cDC were pretreated with CSE for 24 h and then were washed with PBS and treated with 10 µg/mL anti-CCL3 or 0.1 µg/ml anti CXCL2 antibodies or control IgG antibodies for 30–60 min at 37°C. Thereafter, cells were subjected to MLR as described before.

### Statistical analysis

Experimental results are expressed as mean±S.E.M. Results were tested statistically by an unpaired two-tailed Student's t-test or one-way ANOVA, followed by Newman–Keuls test for comparing all pairs of groups. Analyses were performed by using GraphPad Prism (version 4). Results were considered statistically significant when p<0.05.

## Results

### CSE induces CCL3 and CXCL2 production by cDCs

**Figure 1:**
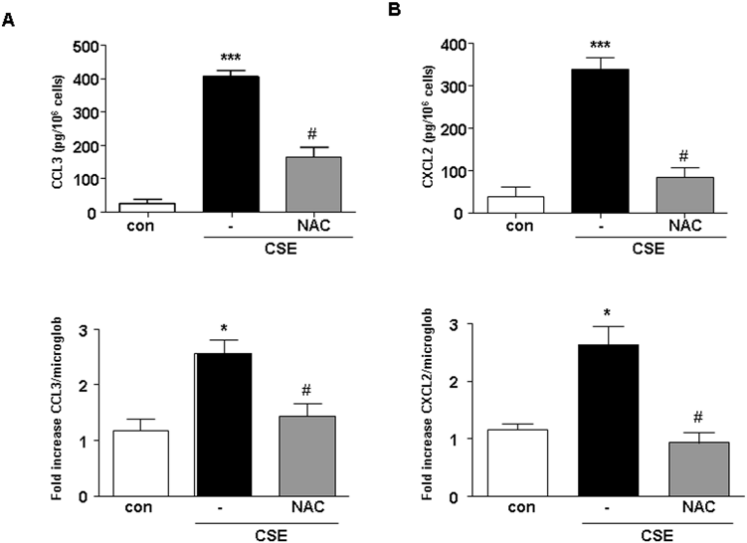
CSE induces the expression of mRNA and the production of chemokines in cDCs. The supernatants of CSE-exposed cDCs were tested for the production and release of CCL3 (A) and CXCL2 (B) ELISA (upper panels) and cell pellets were tested for CCL3 and CXCL2 mRNA levels by real time PCR (upper panels). White bars represent cDCs treated with medium, black bars represent cDCs treated with CSE and gray bars cDCs treated with NAC and CSE. Data are representative of three independent experiments, showing the means±SEM from triplicate cultures. * represents significant differences compared with medium-treated cells (*p<0.05; ***p<0.001). ^#^ indicates significant differences between cells treated with CSE in combination with NAC and cells treated with CSE.

CSE dose dependently (0.035–2.5%) induced the release of chemokines (data not shown). The CSE concentration of 1.5% is most effective in inducing chemokines release from cDCs (Fig. 1A and B, upper panels). Therefore, this concentration was used in all subsequent experiments. Stimulation of cells with CSE (1.5%) did not induce significant TNF-α, IL-2, IL-6, IL-10, IL-12p70, MCP-1 and IFN-γ production (data not shown). CSE-induced CCL3 and CXCL2 production is associated with an increased in mRNA levels for both chemokines (Fig. 1A and B, lower panels). To investigate the involvement of ROS and oxidative stress in the production of CCL3 and CXCL2 by CSE-exposed cDCs, the effect of the antioxidant agent N-Acetyl-Cysteïne (NAC 2.5 mM) was investigated.

**Figure 2:**
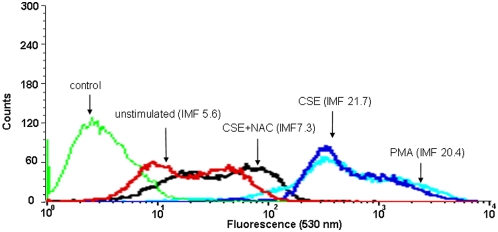
CSE increases the production of intracellular ROS in cDC. cDCs were incubated with CSE , with or without NAC or PMA (as a control) and ROS generation was assayed by FACS analysis. The mean fluorescent intensity (MFI) of the following groups are indicated in the figure: control: unlabelled CSE-treated cells (green line), unstimulated: control labeled cells (red line), CSE: CSE-stimulated labeled cells (blue line), CSE+NAC: CSE-stimulated labeled cells treated with NAC (black line), PMA: PMA-stimulated labeled cells (light blue line).

NAC attenuated the production of CCL3 and CXCL2 induced by CSE (Fig. 1A and B). Furthermore, intracellular ROS production after CSE treatment was measured. Exposure of cDCs to CSE or PMA, as a positive control, resulted in the production of ROS (Fig. 2). Pretreatment of cDCs with the antioxidant NAC resulted in an inhibition of CSE-induced ROS production (Fig. 2).

### TLR4 and MyD88 are involved in the CSE-induced CCL3 and CXCL2 production of cDC

**Figure 3:**
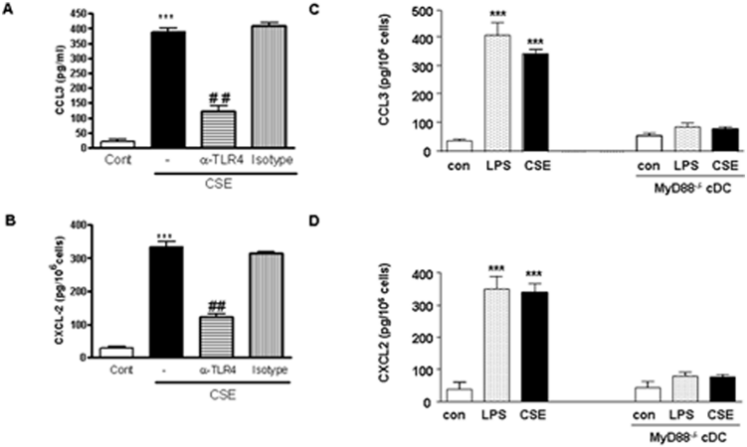
CSE increases the production of CCL3 and CXCL2 by TLR4 and MyD88 dependent manner. cDC were prepared by culturing BM cells from Balb/c mice preincubated with anti-TLR4 antibody (20 µg/ml) or isotype control (20 µg/ml) for 1 h and then stimulated with CSE for 16 h and amount of CCL3 (A) and CXCL2 ( B) were determined by ELISA. cDCs were prepared by culturing BM cells from Balb/c mice and age- and sex-matched MyD88-deficient mice. CSE or LPS were incubated for 16 h and supernatant were harvested for determination of CCL3 (C) and CXCL2 (D) by ELISA. White bars represent cDCs treated with medium, dotted bars are cDCs treated with LPS and black bars represent cDCs treated with CSE. Data are representative of three independent experiments, showing the means±SEM from triplicate cultures. * represent significant differences compared with medium-treated cells (***p<0.001).

We and others have demonstrated that CSE activates inflammatory cells via TLRs [16], [23]. By using neutralizing antibody against TLR4, the releases of CCL3 and CXCL2 was decreased (Fig. 3A and B).

MyD88 is a critical adapter molecule for the transduction of TLRs signals [24]. Therefore, cDCs lacking MyD88 were investigated. CSE did not induce the release of CCL3 and CXCL2 from cDCs obtained from MyD88 knockout mice (Fig. 3C and D). Similarly, LPS, the positive control did not induce a response in MyD88 ^−/−^ cDCs (Fig. 3C and D).

### Involvement of MAPKs and NF-κB in CSE-induced CCL3 and CXCL2 release by cDCs

**Figure 4:**
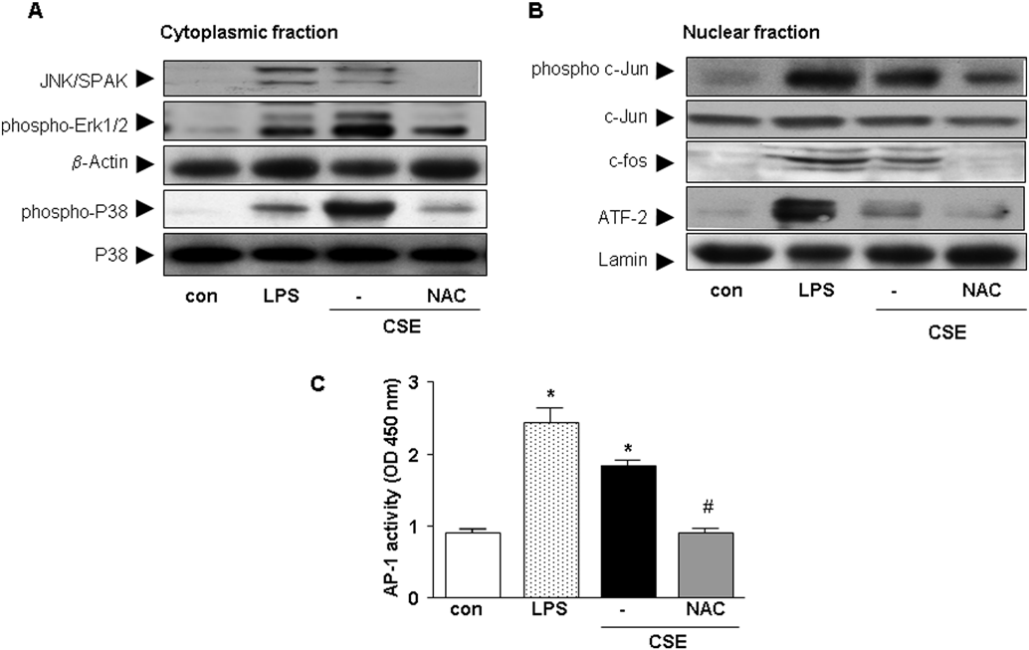
CSE increases the activity of the MAPK pathway in cDCs. Western blot analysis of the cytoplasmic fraction (A) for JNK/SAPK, p-Erk1/2 and p- p38 and p38 from whole cell extracts and of the nuclear fraction (B) ATF-2, p-c-jun, c-jun and c-fos. Representative results of three independent experiments are shown. β-actin and lamin served as loading controls from cytoplasmic and nuclear fractions, respectively. AP-1 activity after stimulation of cells with CSE, LPS or CSE and NAC (C). Values (mean±SEM) are representative data from one of five independent sets of experiments. * indicates significant differences between medium-treated cells and cells treated with LPS or CSE (* p<0.05) and ^#^ represents the significance between cells treated with CSE in combination with NAC and cells treated with CSE (^#^ p<0.05).

It has been reported that CSE activates MAPK and NF-κB in many inflammatory cells [25]–[27]. Therefore, in the current study, the involvement of these pathways were investigated. CSE stimulated phosphorylation of the JNK/SAPK, Erk1/2 and p38 pathways in cytoplasm of cDCs (Fig. 4A). In addition, NAC abrogated the phosphorylation of all these molecules. Next, the effect of pharmacological inhibitors were examined on chemokine release after CSE stimulation. Inhibition of p38 MAP kinase by SB 239063 induced a 63%±17 and 43%±31 reduction of CSE-induced CCL3 and CXCL2 production by cDCs, respectively. Inhibition of Erk1/2 by PD98059 induced a 28.4%±2 and 29%±16 reduction in CCL3 and CXCL2 production, respectively. In the nuclear fraction, CSE increased the phosphorylation of c-jun, c-fos and ATF-2 in cDC (Fig. 4B) and addition of NAC abrogated the phosphorylation of these molecules (Fig. 4B).

CSE and LPS (the positive control) significantly increased the activity of AP-1 compared to control. Pre-incubation of cells with NAC, suppressed the activation of AP-1 induced by CSE (Fig. 4C).

**Figure 5:**
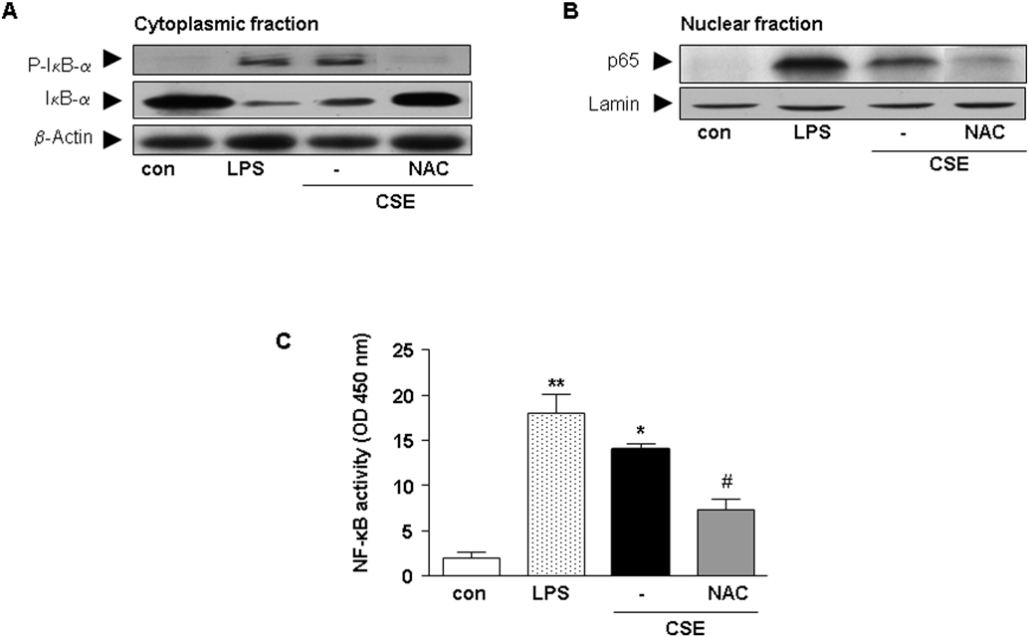
CSE increases the activity of the NF-κB pathway in cDCs. Western blot analysis of the cytoplasmic fraction (A) for IκB-α and p-IκB-α from whole cell extracts and of the nuclear fraction (B) p65 were carried out with related antibodies. Representative results of three independent experiments are shown. β-actin and lamin served as loading controls from cytoplasmic and nuclear fractions, respectively. NF-κB activity after stimulation of cells with CSE, LPS or CSE and NAC. (C). Values (mean±SEM) are representative data from one of five independent sets of experiments. * Indicates significant differences between medium-treated cells and cells treated with LPS or CSE (* p<0.05) and ^#^ represents the significance between cells treated with CSE in combination with NAC and cells treated with CSE (^#^ p<0.05).

Further, the regulation of NF-κB signaling in cDCs by CSE was investigated. To address the mechanism involved in the degradation of IκB-α by CSE, phosphorylation of IκB-α by Western blot analysis was examined. CSE or LPS (the positive control) increased IκB-α phosphorylation (Fig. 5A) and exposure to CSE or LPS resulted in the degradation of IκB-α (Fig. 5A). Pretreatment with NAC inhibited the CSE-induced phosphorylation and degradation of IκB-α (Fig. 5A). In the nuclear fraction CSE and LPS increased the nuclear translocation of p65 which was abrogated by NAC (Fig. 5B). Pretreatment of cDC with the pharmacological NF-κB inhibitor (curcumin) resulted in an 80%±3 reduction of the chemokine production (data not shown). For determination the activity of NF-κB, nuclear proteins were subjected to a reaction containing biotin conjugated-oligonuclotides NF-κB. CSE increased the activity of NF-κB in cDCs and pretreatment with NAC suppressed NF-κB activity (Fig. 5C).

### CSE-primed cDCs stimulate the proliferation of CD8^+^T cells

**Figure 6:**
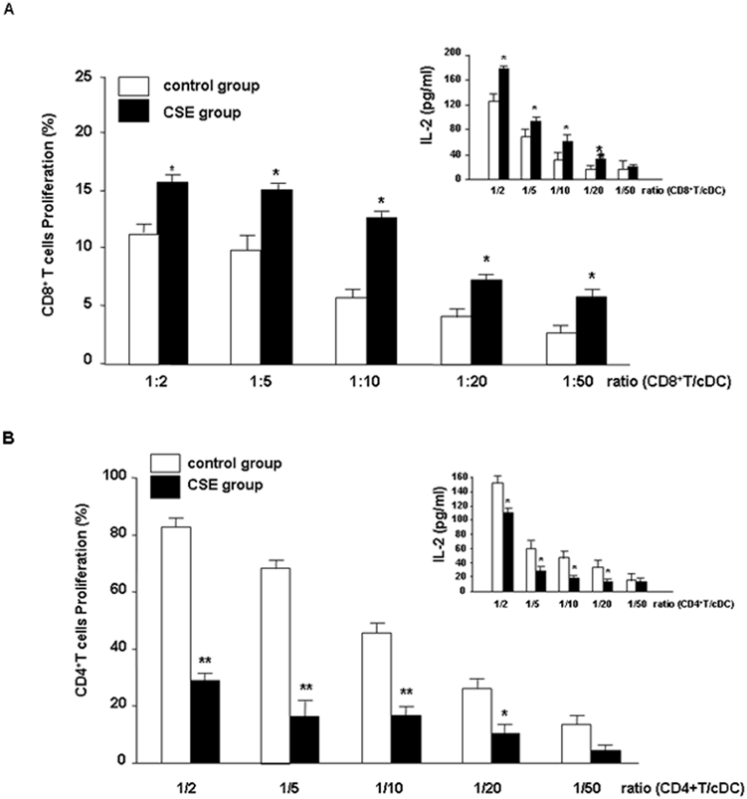
CSE increases cDC-induced CD8^+^T cell but inhibits CD4^+^T cell proliferation. cDCs from Balb/c mice were incubated with medium (white bars) or CSE ( black bars) and coincubated with allogenic T cells from C57BL/6 mice [CD8^+^ (A) and CD4^+^T cells (B)] in a MLR. Presented are pooled data from eight individual experiments using cDCs generated from eight isolations. Values are represented as mean±SEM. A statistically significant modulation of proliferation of T cells with CSE-primed cDCs occurred (^*^ *p*<0.05 and ** p<0.01 when compared to medium-treated cDCs). The supernatants of MLR were collected for the measurement of IL-2 by ELISA (inserted graphs in A & B). Presented are pooled data from eight individual experiments using cDCs generated from eight isolations. Values are represented as mean±SEM. * Indicates significant differences between medium-treated cells (* p<0.05).

CSE significantly increased the ability of cDCs to stimulate the proliferation of CD8^+^T cells compared with untreated cDCs (Fig. 6A) and decreased proliferation of CD4^+^T cells (Fig. 6B). In a reciprocal fashion, the MLR was also determined by using T cells exposed to CSE. CSE did not effect proliferation of T cells (CD4^+^ or CD8^+^T cells) when co-cultured with untreated allogeneic cDCs (data not shown). To further characterize the effect of CSE on the priming capacity of cDCs, the effect of CSE on IL-2 production in the MLR was assessed. As depicted in Fig 6A upper panels, IL-2 production of CD8^+^T cells was increased and decreased by CD4^+^T cells (Fig. 6B, upper panel).

### CCL3 antibodies suppress CSE-primed cDC- induced proliferation of CD8^+^T cells

Finally, the involvement of CCL3 and CXCL2 in MLR reaction were investigated.

**Figure 7:**
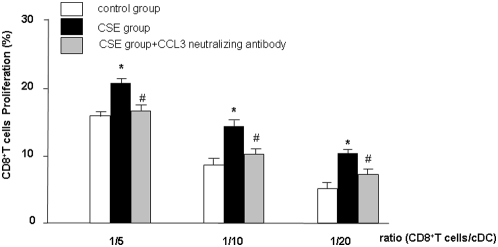
CCL3 neutralizing antibodies suppresses CSE-primed cDC-induced proliferation of CD8^+^T cells. cDCs were incubated with medium (white bars) or CSE and then incubated without (black bars) or with polyclonal antibodies neutralizing CCL3 (gray bars). The cDCs were co-cultured with CD8^+^T cells. Data represent means±SEM of triplicate experiments. * represents significant differences compared with medium-treated cDCs (*p*<0.05, n = 3). # indicates significant differences between CSE-primed cells and CCL3- treated and CSE-primed cells.

cDCs were exposed with CSE for 24 h, treated with neutralizing antibodies directed against CCL3 or CXCL2 and were then subjected to MLR reaction. Incubation of neutralizing CCL3 antibody in MLR, profoundly suppressed the CSE-primed cDC–induced proliferation of CD8^+^T cells in the MLR (Fig. 7). Incubation with CCL3 antibody did not affect the CSE-primed cDCs-induced reduction of CD4^+^T cell proliferation (data not shown). Moreover, the effects of CXCL2 neutralizing antibodies on the MLR with CSE-primed cDCs and CD8^+^ or CD4^+^T cells were investigated. CXCL2 antibody had no effect on the proliferation of CD8^+^T cells or CD4^+^T cells when co-cultured with CSE-primed cDCs (data not shown).

## Discussion

In this study, the effects of CSE on cDCs were explored with particular emphasis on the function and cellular immune responses. Among the tested cytokines and chemokines, CSE induced the release of CCL3 and CXCL2 by a ROS dependent manner. Interestingly, CSE did not induce the production of TNF-α, IL-2, IL-6, IL-10, IL-12p70, MCP-1, IFN-γ and even suppressed the production of these cytokines induced by LPS (data not shown). Similar data on IL-12 and IL-23 have been published by Kroening et al, [28]. Our findings are consistent with the work of others showing the induction of IL-8 by CSE or cigarette smoke in human pulmonary DCs and in an in vivo model of smoke exposed mice [29].

Moreover, we show for the first time that CSE modulates cDC-mediated of T cells and specifically augments proliferation of CD8^+^T cells and inhibits proliferation of CD4^+^T cells in MLR. The increase in proliferation of CD8^+^T cells is mediated by CCL3, since the increase in proliferation is inhibited by antibodies against CCL3. CXCL2 antibodies did not have an effect (data not shown).

Cigarette smoke contributes to oxidant-induced damage of the cells via oxidants and free radicals [30] and generation of intracellular ROS [31]. We showed, that CSE induces ROS production in cDCs leading to the production and release of CXCL2 and CCL3. Interestingly, the generation of chemokines by cigarette smoke-activated DCs could be mitigated by anti-oxidants, NAC treatment. These data indicate that anti-oxidant therapy with agents like NAC may effect cigarette smoke-induced chemokine release of cDCs.

Next we found that MyD88/TLR4 activation and NF-κB/MAPK signaling is involved in the induction of chemokines by CSE in cDCs. The first signaling protein to be recognized as oxidative stress-sensitive molecules are transcription factors, such as NF-κB [32] . ROS strongly affects the activation of NF-κB [32]. Besides, the MAPK pathway is an important signaling pathway affected by CSE [26]. In the current study, CSE induces the release of chemokines by both the activation of NF-κB and the MAPK pathways since inhibition of these intracellular signaling pathways suppresses the release of both chemokines. Chemokines regulate the movement of leukocytes such as neutrophils and lymphocytes [33]. The predominant chemokine for human neutrophils is the CXC chemokine CXCL8. Mice lack CXCL8 but have the neutrophilic CXC chemokine ligand 2, MIP-2 or CXCL2 [34]. The importance of this chemokine in promoting pulmonary inflammation associated with COPD has extensively been investigated in vitro and in vivo [35]–[37]. Therefore, the CSE-induced release of CXCL2 by cDCs may result in the infiltration and activation of neutrophils in the airways.

Interestingly, in current study we show that CSE-primed cDCs increase the proliferation of CD8^+^ and suppress CD4^+^T cells proliferation. In the supernatants of MLR samples the IL-2 production is elevated in CD8^+^T cells which is in agreement with the proliferation of cells. These data could explain the enhanced CD8^+^T cell population observed in lungs of smokers and smoke-treated mice [37]–[39]. Until now, the mechanism for this process is not well documented. Maeno and coworkers, described a critical role for CD8^+^T cells in inflammatory cell recruitment and lung destruction in a cigarette smoke-induced murine model for COPD [12]. Earlier evidence reported that CCL3 is involved in CD8^+^T cell proliferation [40]. Interestingly, CCL3 production by cDCs after CSE stimulation has a central role in the induction of the proliferation of CD8^+^T cells since proliferation was blocked by adding CCL3 neutralizing antibody. Moreover, CSE-primed cDCs suppress CD4^+^T cells proliferation which is agreement with recent *in vivo* studies [38]. The role and amounts of CD4^+^T cells in COPD is not well documented but early studies reported that cigarette smoke exposure led to a specific decrease in the percentage of activated CD4^+^T cells, but not CD8^+^T cells in the lung [37]. Interestingly, very recently, Harissison et al reported that the total number of BAL CD4^+^ and CD8^+^T cells is higher in mice exposed to cigarette smoke. Furthermore, CD4^+^T cells were proportionally higher than CD8^+^T cell [41]. We tested the effects of neutralization antibodies against CCL3 and CXCL2 on the decreased CD4^+^ T cell proliferation induced by CSE-primed cDCs and did not find any suppressive effects on (data not shown). The reasons for the above mentioned discrepancies are not clear. The decrease in proliferation of CD4^+^T cells may indicate suppressive effects of cigarette smoke on immune responses and may account for the higher susceptibility of smokers to viral and bacterial infections [42], [43].

The above-mentioned explanation for the regulation of proliferation of T cells by CSE primed-DCs is an over simplification and is mainly used as a working hypothesis. In summary, cigarette smoke induces the release of CXCL2 and CCL3 by cDCs. CXCL2 is considered as a chemokine that can recruite neutrophils. CCL3 results in the proliferation of CD8^+^T cells and may be a key factor for increasing this cell in smokers and COPD patients. However, the relevance of above mentioned data should be confirmed in animal model with COPD and human.
